# 

**Published:** 1940

**Authors:** 


CONTAINS INDEX

INDEXED

Vol. LVII. No. 217. WINTER, 1940.

### The Bristol Medico-Chirurgical Journal

ARMY MEDICAL MAY 12 1941 LIBRARY

PUBLISHED UNDER THE AUSPICES OF

THE BRISTOL MEDICO-CHIRURGICAL SOCIETY.

*Editor:* J. A. NIXON, C.M.G., M.D., F.R.C.P.

WITH WHOM ARE ASSOCIATED

E. W. HEY GROVES, D.Sc., M.S., F.R.C.S.

A. E. ILES, O.B.E., M.B., B.S., F.R.C.S.

A. RENDLE SHORT, M.D., B.S., B.Sc., F.R.C.S.

W. MELVILLE CAPPER, M.B., B.S., F.R.C.S.

T. M. LING, M.D., M.R.C.P.

E. WATSON-WILLIAMS, M.C., Ch.M., F.R.C.S., *Assistant Editor.*

BRISTOL:

J. W. ARROWSMITH LTD., QUAY STREET & SMALL STREET

Price Three Shillings net.

